# Establishment of a novel alloxan‐induced rabbit model exhibiting unique diabetic retinal neuropathy features assessed via ERG + VEP

**DOI:** 10.1002/ame2.70032

**Published:** 2025-06-19

**Authors:** Xinlu Li, Xiaojing Dong, Defei Feng, Han Hu, Bai Li, Zhongjian Liu, Wei He, Chenchen Huang, Zhizhou Shi, Yan Mei

**Affiliations:** ^1^ Faculty of Life Science and Technology Kunming University of Science and Technology Kunming China; ^2^ Department of Ophthalmology The Affiliated Hospital of Kunming University of Science and Technology Kunming China; ^3^ Department of Ophthalmology The First People's Hospital of Yunnan Province Kunming China; ^4^ Medical School Kunming University of Science and Technology Kunming China; ^5^ Center for Clinical Medicine Research The First People's Hospital of Yunnan Province Kunming China

**Keywords:** alloxan, diabetic retinal neuropathy, ERG+ops, rabbit, VEP

## Abstract

**Background:**

Diabetic retinal neuropathy (DRN) leads to significant visual impairment; however, no existing animal model fully replicates its neural alterations, and inconsistent induction protocols with high mortality rates hinder long‐term investigations.

**Methods:**

Adult male rabbits were randomly assigned to four experimental groups, each receiving a single intravenous injection of varying doses of alloxan and one control group. The safety and efficacy of alloxan in inducing diabetes were evaluated to determine the optimal dose. At 9 weeks following injection with alloxan, retinal function was assessed using full‐field electroretinography (ERG) and visual evoked potentials (VEPs). Retinal structure was examined in rabbits using spectral‐domain optical coherence tomography (SD‐OCT), Optos ultra‐widefield (Optos UWF) false‐color imaging, and widefield fundus fluorescein angiography (WF‐FFA).

**Results:**

Rabbits in the 80 mg/kg alloxan group exhibited fewer complications, lower mortality, and a higher model success rate compared to other groups. At 9 weeks post‐injection, these rabbits demonstrated significantly elevated hemoglobin A1c and total cholesterol (*p* < 0.05) relative to controls. ERG revealed statistically significant reductions in oscillatory potential and b‐wave amplitudes (*p* < 0.05), while VEP indicated decreased P2 amplitude (*p* < 0.001) and prolonged P2 latency (*p* < 0.05). SD‐OCT, Optos UWF imaging, and WF‐FFA demonstrated no significant changes in vascular abnormalities. Additionally, Hematoxylin and Eosin staining revealed retinal swelling (*p* < 0.05), and immunofluorescence confirmed glial activation and neuronal loss.

**Conclusions:**

A single intravenous injection of 80 mg/kg alloxan effectively and safely induced DRN in rabbits, resulting in neural retina damage, thereby establishing this model as an ideal model for DRN research.

## Graphical abstract

Study workflow. Neuroretinal changes were observed in alloxan‐induced diabetic rabbits, while the retinal vasculature remained unaffected, indicating the successful establishment of a diabetic neuroretinal model.

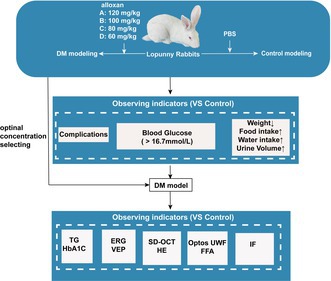

## 1 INTRODUCTION

The number of individuals with diabetic retinopathy (DR) is projected to increase from 126.6 million in 2011 to 191 million by 2030.^1^ Moreover, retinal neuropathy is the earliest characteristic to manifest in DR.^2^, ^3^ The diabetic retinal neuropathy (DRN) pathogenesis involves oxidative stress, mitochondrial dysfunction, chronic inflammation, and glutamate excitotoxicity, which lead to neuronal apoptosis and glial cell activation.^4^ In the early stages, DRN plays a pivotal role in DR pathogenesis, with nerve impairment occurring before vascular harm.^5^ Consequently, DRN significantly contributes to DR and plays a crucial role in the initial onset of vision impairment in patients with diabetes, potentially serving as an indicator of early‐stage retinal damage.^6^ Currently, no animal model fully replicates the complete pathophysiology of neuronal changes occurring at every stage of DRN. Therefore, developing a neuronal animal model for DRN is critical in preventing vision loss in patients with DR.

As our understanding of the complex interactions among neurons, glial cells, and vascular components in DR advances, the demand intensifies for an animal model that accurately replicates the neurological damage of DRN while enabling precise experimental manipulation.^7^, ^8^ Traditional models exhibit significant limitations. For instance, cat models display vascular leakage and neovascularization but are characterized by prolonged induction periods and resistance to STZ.^9^, ^10^ Although dog models possess retinal structures similar to those of humans, they are limited by extended induction times, complex handling requirements, and high maintenance costs.^11^ Similarly pig models, while anatomically comparable to human eyes, are disadvantaged by rapid weight gain, which restricts their use primarily to ex vivo studies.^12^, ^13^ Rodent models offer cost efficiency and are well established; however, their small ocular dimensions and rod‐dominated retinas limit their relevance for investigating human neurodegenerative processes.^14^, ^15^ In contrast, rabbit models were 9 weeks that can effectively replicate early DRN features of DRN.^16^, ^17^ Their larger eye size supports high‐resolution imaging, precise intraocular interventions, and detailed investigation of neurovascular coupling mechanisms.^18^ Furthermore, rabbits have anatomical and physiological retinal characteristics similar to those of humans. With an 18‐mm eye diameter, rabbit ocular structures (cornea, lens, and retina) and retinal cell counts closely resemble those of human eyes. Additionally, their higher cone density in the visual streak, similar to the human macula, allows for more precise observations compared to rodent models.^19^, ^20^

In contrast to rodent models, where cone photoreceptors comprise only approximately 3% of the retina, the rabbit retina has a higher cone proportion, especially in the visual streak, which is analogous to the human macula.^21^, ^22^ This anatomical similarity, combined with their larger eye size, allows for more precise and clinically relevant observations.^23^, ^24^ However, previous diabetic rabbit studies have identified significant drawbacks compromising model reliability and reproducibility. High mortality rates and a lack of detailed information on the induction process and causes of death have been frequently reported, resulting in unstable models.^25^, ^26^ Additionally, although some studies report fundus lesions such as retinal hemorrhage, the lack of detailed descriptions, particularly regarding electroretinography (ERG) waveform changes, compromises experimental accuracy.^27^, ^28^, ^29^ Variability in induction methods further complicates inter‐study comparisons and replication.^17^, ^21^, ^30^, ^31^ Moreover, the dense distribution of retinal blood vessels in rabbits hinders direct observation of vascular lesions, and inconsistent evaluation techniques limit the models' translational relevance.^32^, ^33^ In contrast, this study improves model stability and reproducibility by standardizing the induction process, reducing mortality, employing advanced techniques such as fundus angiography and optical coherence tomography (OCT), and offering detailed ERG and pathological change analyses. This approach provides more reliable and relevant data for understanding human ocular conditions.

Mature modeling standards for the DRN model have not yet been established. Developing reliable and robust methods to assess neuronal damage in diabetes is essential for designing effective experiments and preventing irreversible, clinically significant retinopathy. For this purpose, we established a model characterized by (i) neuronal retina abnormalities induced by diabetes, while vascular abnormalities remained unaffected, and (ii) evidence suggesting that clinical methods can detect retinal neuropathy and nerve damage at early stages.^34^, ^35^, ^36^

This study determined the optimal intravenous alloxan dose for inducing diabetes in rabbits and introduced a novel approach by integrating retinal structure and function analyses in a DRN model. ERG, oscillatory potentials (OPs), and visual evoked potentials (VEPs) were combined to comprehensively evaluate retinal function and changes in the central visual pathway. Additionally, retinal structure and vascular imaging in awake DRN rabbits were assessed using spectral‐domain OCT (SD‐OCT), Optos ultra‐widefield imaging (Optos UWF), and widefield fundus fluorescein angiography (WF‐FFA). Histopathological changes in retinal nerve structure and cellular components were analyzed through Hematoxylin and Eosin (H&E) staining and immunofluorescence (IF). This innovative diabetic model of retinal neuropathy provides a critical foundation for future studies and quantitative clinical diagnostic assessment.

## 2 METHODS

### 2.1 Animals

**FIGURE 1:**
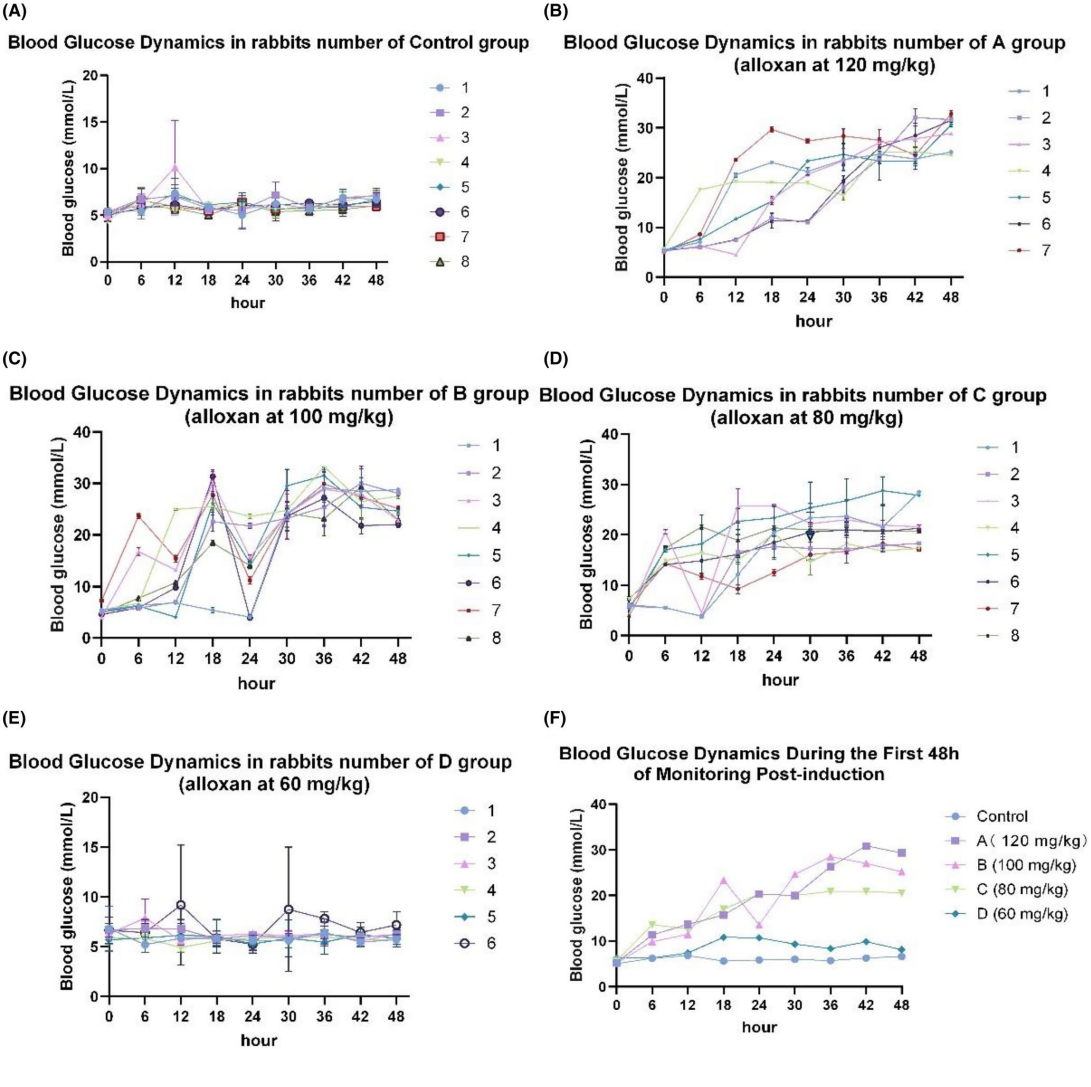
Graphical representation of blood glucose dynamics in individual rabbits from control and alloxan‐treated groups during the first 48 h post‐induction. (A) Control group blood glucose dynamics (*n* = 8). (B) Blood glucose dynamics in group A (120 mg/kg, *n* = 7). (C) Blood glucose dynamics in group B (100 mg/kg, *n* = 8). (D) Blood glucose dynamics in group C (80 mg/kg, *n* = 8). (E) Blood glucose dynamics in group D (60 mg/kg, *n* = 6). (F) Blood glucose dynamics in all individual rabbits across the five groups. Each number represents an individual rabbit analyzed under identical experimental conditions. Rabbits were assigned unique identification number within their respective group: Control group (1–8), group A (120 mg/kg, 1–7), group B (100 mg/kg, 1–8), group C (80 mg/kg, 1–8), and group D (60 mg/kg, 1–6). Data reflect individual rabbit measurements grouped according to treatment conditions.

Eight‐week‐old male rabbits (2.0–2.5 kg) bred at the Animal Experiment Center of Kunming Medical University were housed at 21°C under a 12 h light/dark cycle with 60% humidity. Rabbits were given ad libitum access to a standard pellet diet, fresh carrots, and water. Before modeling, the animals underwent a 12‐h fast and were permitted food and water 48 h post‐procedure. For anesthesia, rabbits weighing 2.00–3.00 kg were administered intramuscular xylazine (5 mg/kg), followed 5–10 min later by intravenous ketamine (80 mg/kg). The cessation of heartbeat and respiration confirmed anesthesia, and no clinical signs of distress were observed. Diabetes was induced via intravenous injection of alloxan (Sigma Aldrich). At the study endpoint, the animals were humanely euthanized using an overdose of anesthetics. The study design is illustrated in the Graphical Abstract. As described in previous studies,^37^, ^38^, ^39^ animals were randomly assigned to four experimental groups: A (alloxan 120 mg/kg, *n* = 8), B (alloxan 100 mg/kg, *n* = 8), C (alloxan 80 mg/kg, *n* = 8), D (alloxan 60 mg/kg, *n* = 6), and one control group (*n* = 8). In group D, all six rabbits did not exhibit a significant increase in blood glucose levels (BGLs) following alloxan administration, which was considered sufficient for statistical analysis. No additional animals were included, given the consistent results, ethical considerations, literature precedent,^40^, ^41^ and resource optimization. Rabbits in the control group received 10 mL/kg physiological saline injections. Blood sugar measurements taken throughout the study period are presented in Figure 1. As described in previous studies,^23^, ^42^ glucose levels were measured using an Accu‐Chek blood glucose meter (Accu‐Chek, Roche Diabetes Care) within 48 h post‐alloxan injection and subsequently every 6 h until hyperglycemic conditions were confirmed. Rabbits with BGLs exceeding 16.7 mmol/L (300 mg/mL) were selected in two separate assessments.^43^, ^44^

### 2.2 Biochemical measurements in the plasma and serum

Rabbits were anesthetized with intramuscular xylazine (3 mg/kg), followed by intravenous ketamine (25 mg/kg) 5–10 min later. A 1 mL blood sample was collected from the ear vein and transferred into plain or ethylenediaminetetraacetic acid tubes (2 mL capacity). Plasma hemoglobin A1c (HbA1c) levels were quantified using the Cobas c501 module (Roche Diagnostics), while serum total cholesterol (TC) was measured using immunoturbidimetry. Analyses were performed on 22 samples per group using rabbit‐specific kits following the manufacturer's protocols.

### 2.3 ERG

Each rabbit received 0.5% tropicamide eye drops for 30 min under dark‐adapted conditions to achieve full pupil dilation. Corneal fabric electrodes were applied, with reference skin electrodes placed on the temporal aspects of each eye and copper counter electrodes on the earlobes. Recordings adhered to the International Society for Clinical Electrophysiology of Vision standards for clinical multifocal ERG (2022 edition). Both eyes were assessed using dark‐adapted 3.0 ERG + OPs and 10.0 ERG + OPs protocols. The ESPION ERG system (Diagnosys, Littleton, USA) automatically quantified the number, delay, and mean amplitude of wavelets (a, b, OP1, OP2, OP3, and OP4). The same experienced technicians conducted examinations, and baseline and 8‐week data were analyzed. Three consecutive stable waveforms confirmed the repeatability of the measurements.

### 2.4 Flash‐VEP (F‐VEP)

F‐VEP responses were elicited by a brief, intense light stimulus covering a minimum 20° visual field. Scalp electrodes, placed according to the International 10/20 System, recorded a series of positive and negative waves typically occurring between 30 and 300 ms, with N2 and P2 waves, at approximately 90 and 120 ms, being the most prominent. This stimulation protocol is highly suited for animal studies.

### 2.5 UWF Optos fundus imaging

Optos UWF enhances peripheral retinal visualization. Retinal images were acquired in awake rabbits using the Daytona (P200T, OPTOS, UK) equipped with a universal retinal lens (biogenic field of vision) at a resolution of 14 μm, both before and after induction. Five high‐quality images per eye were selected, capturing the detailed retinal vascular network with exceptional precision.

### 2.6 SD‐OCT image acquisition

Images were obtained from awake animals with minimal manual restraint by gently holding and wrapping them in a towel. Retinal and optic neuroimaging of all animals was performed using axis pixels with a universal retinal lens (biogenic field of vision) at a resolution of 2.4 μm (Spectralis OCT, Germany).

### 2.7 WF‐FFA

WF‐FFA was performed under general anesthesia using a confocal laser scanning angiography system (Spectralis HRA, Heidelberg, Germany). Rabbits were positioned on an examination table with their heads stabilized on a jaw rest supported by a soft cushion. Eyelids were retracted with a lid speculum, and pupil dilation was confirmed. Following rapid fluorescein sodium injection, imaging began immediately, capturing the posterior pole and the supratemporal, infratemporal, supranasal, and inferonasal infranasal fundus quadrants.

### 2.8 HE staining

Following euthanasia, the rabbits' eyes were enucleated and fixed in 4% paraformaldehyde (Wuhan Sevier Biological Co., Ltd) for 24 h and dehydrated using a graded series of alcohols. The anterior segment and vitreous body were removed, and the remaining eye cup was bisected along the vertical meridian to preserve the tissue surrounding the optic nerve. Specimens were initially embedded in 2% agar to prevent retinal separation before paraffin embedding. The entire retina was sectioned vertically through the optic nerve into 5 μm slices and stained with H&E for routine histological analysis.

### 2.9 Immunofluorescence staining

Tissue sections were dewaxed in 100% xylene, rehydrated through graded ethanol, and rinsed in phosphate‐buffered saline. Antigen retrieval was performed in citrate buffer (pH 6; Vector Laboratories) by heating for 20 min and allowing the sections to cool for 20 min at room temperature. Blocking was performed for 1 h at room temperature using 0.01% Triton X‐100 and 10% donkey or goat serum. Primary antibodies against Glial Fibrillary Acidic Protein (GFAP) (GB11096‐50; Servicebio) and RNA‐binding protein with multiple splicing (RBPMS) (MG770204; Abmart) were incubated overnight at 4°C, followed by a 2‐h incubation with secondary antibodies at room temperature. Finally, nuclei were counterstained with DAPI (G1012‐100ML; Servicebio).

### 2.10 Statistical analyses

The statistical analyses were conducted using the GraphPad Prism software (version 10.0). A Student's *t* test with a two‐tailed distribution was employed to assess statistically significant differences between the average values of groups. Results were considered statistically significant when *p* ≤ 0.05. Data are presented as mean ± standard deviation (SD).

## 3 RESULTS

### 3.1 Safety and efficacy evaluation of different doses of alloxan‐induced diabetes in rabbit

#### 3.1.1 Blood glucose dynamics and survival rates in rabbits 48 h after administration of different alloxan doses

As illustrated in Figure 1, the baseline BGLs of the rabbits were within the normal range (5–7.5 mmol/L) following an 8–12 h fast. The rabbits were randomly assigned to five experimental groups (Figure 1A–E): A (120 mg/kg), B (100 mg/kg), C (80 mg/kg), D (60 mg/kg), and Control. Alloxan was administered in a single injection into the lateral ear vein at varying doses. BGLs increased in diabetic rabbits 1 h after alloxan injection. This initial phase peaked at approximately 6 h following the alloxan injection, with most rabbits reaching an average blood glucose of 10.10 ± 5.53 mmol/L.

Subsequently, BGLs gradually decreased. Four rabbits, including one from Group A (120 mg/kg, No. 3), one from Group B (100 mg/kg, No. 5), and two from Group C (80 mg/kg, Nos. 1 and 6), reached a minimum BGL of 3.8–3.9 mmol/L following 12 h of alloxan administration (Figure 1B–D). These rabbits were administered intravenous glucose injections. As depicted in Figure 1F, alloxan‐induced diabetic rabbits exhibited elevated BGLs compared to the control group. Successful modeling was confirmed in rabbits with BGLs ≥16.7 mmol/L (or ≥350 mg/dL). However, the BGLs of the rabbits in group D (60 mg/kg) remained unchanged. Group A (120 mg/kg) exhibited the highest BGLs at 29.28 ± 1.47 mmol/L, indicating a positive correlation between the mean BGL and the administered dose. Group C (80 mg/kg) steadily increased BGLs, reaching 20.05 ± 7.79 mmol/L. During the 48‐h monitoring period, one rabbit in group A (120 mg/kg) died, while 37 rabbits survived. In diabetic rabbits, alloxan (80 mg/kg) effectively induced blood glucose elevation with minimal side effects.

#### 3.1.2 Metabolic changes and diabetes‐related symptoms in rabbits 6 weeks after alloxan injection at different doses

**FIGURE 2:**
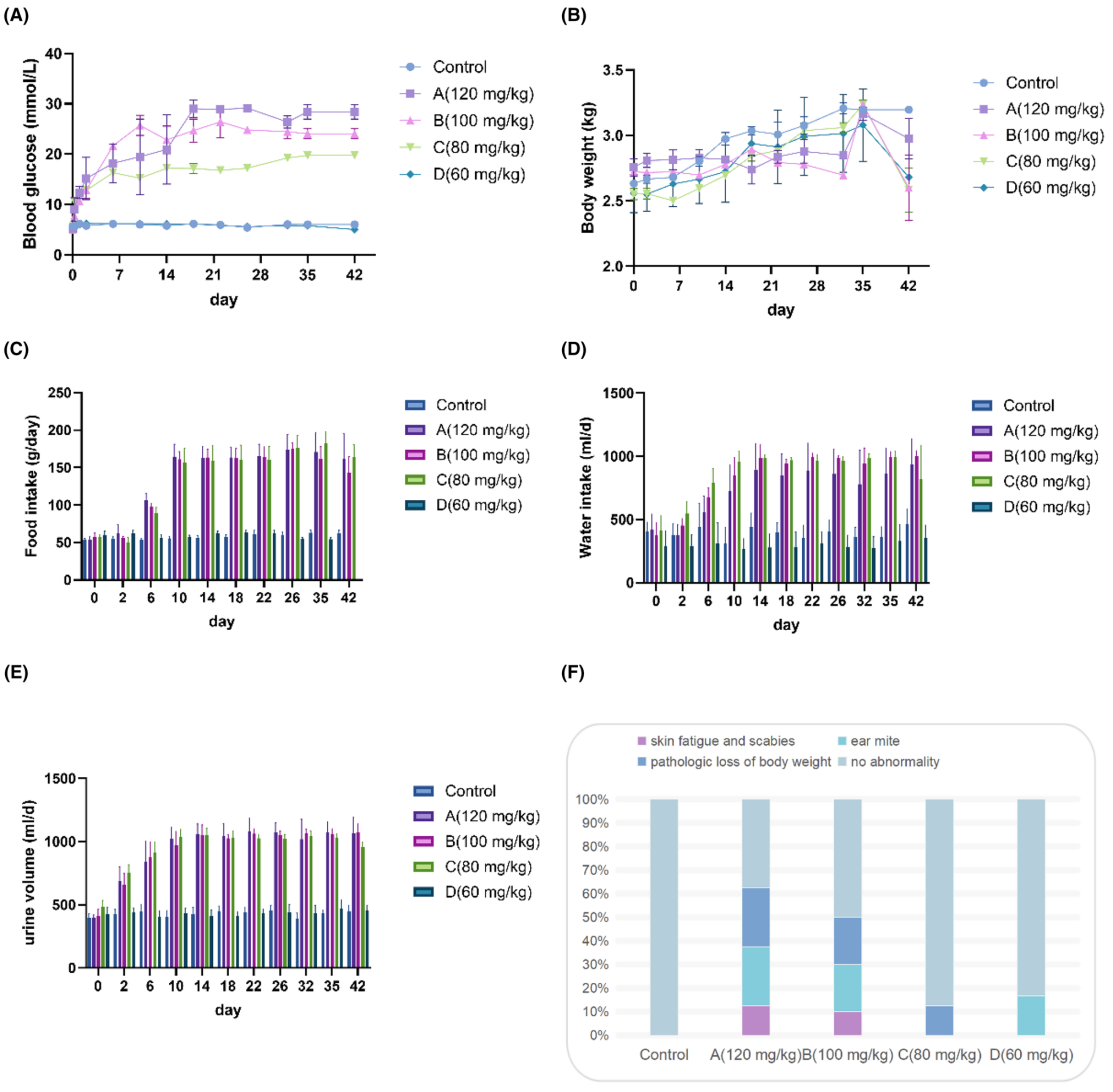
Effects of alloxan on blood glucose, body mass, and diabetes‐related characteristics in the experimental rabbit groups. (A) Weekly trends in BGLs following injection. (B) Weekly trends in body mass weeks post‐injection. (C–E) Weekly food intake, water intake, and urine volume trends, respectively. (F) Comparison of diabetes‐related complications among rabbits treated with different alloxan doses: A group (120 mg/kg), B group (100 mg/kg), C group (80 mg/kg), and D group (60 mg/kg). Data are presented as the mean ± SD.

Body weight, BGLs, body mass, and diabetes‐related characteristics were assessed in the control and alloxan‐treated groups, which received varying doses of alloxan. Significant increases in the BGLs were observed in the groups A (120 mg/kg), B (100 mg/kg), and C (80 mg/kg) following alloxan injection in normal rabbits (Figure 2A). No change was detected in the D group (60 mg/kg). However, group A (120 mg/kg) exhibited higher BGLs compared to groups B (100 mg/kg) and C (80 mg/kg). Group B (100 mg/kg) exhibited fluctuation in BGL, whereas the BGLs of group C (80 mg/kg) displayed a stable trend (Figure 2A).

Following alloxan injection, an overall increase in body weight was observed in all four groups during the first 5 weeks, indicating that rabbit feeding activity remained unaffected. However, after 6 weeks of alloxan injection, a decrease in body weight was noted in groups A (120 mg/kg), B (100 mg/kg), and C (80 mg/kg) (Figure 2B), while continuous weight gain was observed in the control and D (60 mg/kg) groups of rabbits (Figure 2B).

To assess diabetes‐related symptoms, food intake, water intake, and urine volume were elevated in the model groups compared to the control and D (60 mg/kg) groups (Figure 2C–E). Within 1 week post‐induction, groups A (120 mg/kg), B (100 mg/kg), and C (80 mg/kg) reached peak levels of food intake, water intake, and urine volume, with variation corresponding to the alloxan dose. These findings suggested that diabetes‐related characteristics intensified in the high‐dose groups. As presented in Figure 2F, skin fatigue and ear mite infestation were more prominent in groups A (120 mg/kg) and B (100 mg/kg) than in group C (80 mg/kg) rabbits. Furthermore, groups A (120 mg/kg) and B (100 mg/kg) demonstrated a significantly greater pathological body weight loss over the 6 weeks compared to the other groups, with reductions exceeding 20% within 14 days following alloxan administration.

#### 3.1.3 Six weeks survival rates after administration of different alloxan doses in rabbits

**FIGURE 3:**
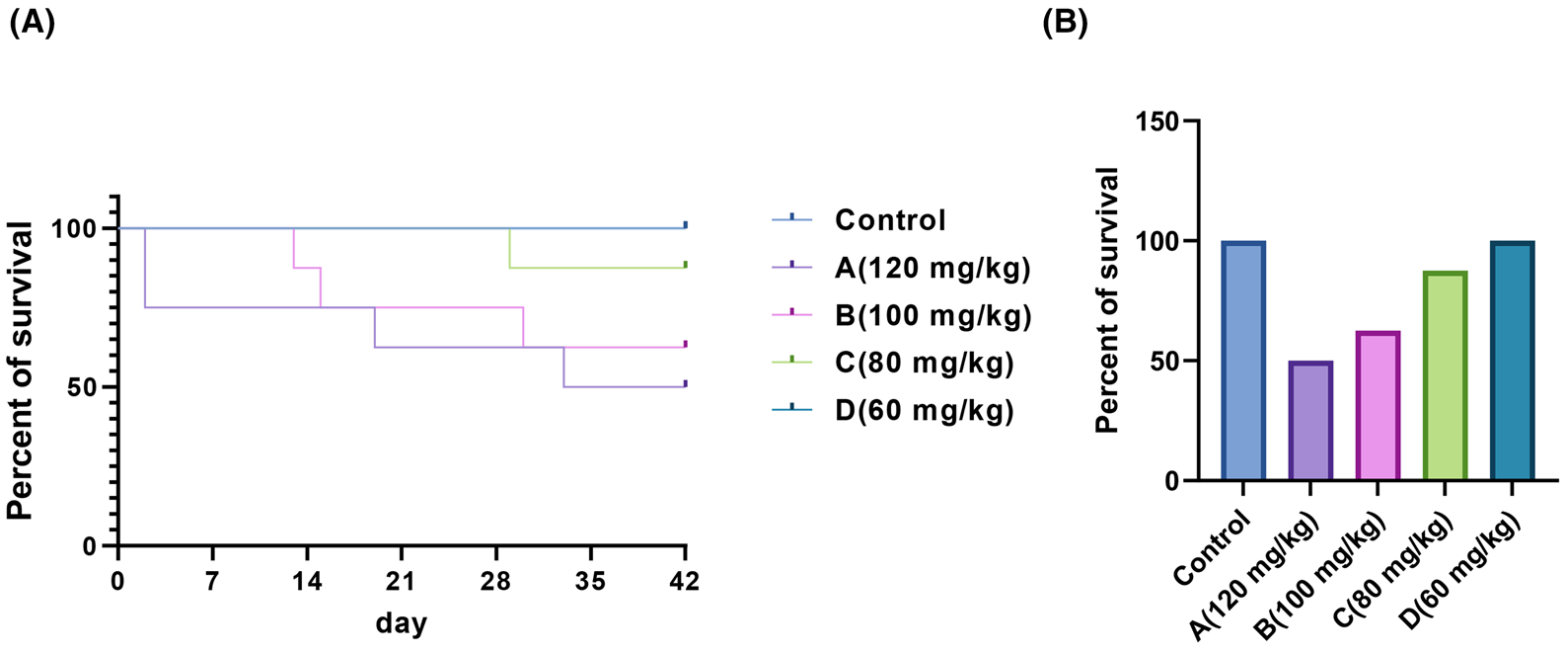
Survival percentage in all groups of rabbits during the 6 week post‐induction. (A) The dynamic trends in mortality rates. (B) The survival rates. A group (120 mg/kg), B group (100 mg/kg), C group (80 mg/kg), and D group (60 mg/kg).

The dynamic trends in mortality and survival rates across all groups are presented in Figure 3A,B. No mortality was recorded in the control and D (60 mg/kg) groups. During disease progression, group A (120 mg/kg) experienced four deaths, resulting in a 6‐week survival rate of 58.3%. Group B (100 mg/kg) recorded two deaths, corresponding to a survival rate of 62.5% at 6 weeks. Among the deceased diabetic rabbits, five deaths were attributed to deteriorating health conditions, while one was caused by alloxan toxicity. In contrast, group C (80 mg/kg) achieved a survival rate of 87.5%, with only one diabetic rabbit dying due to poor health conditions by 6 weeks following alloxan injection. Consequently, these findings suggest that a single injection of 80 mg/kg of alloxan via the lateral ear vein partially improved survival outcomes in diabetic rabbits.

### 3.2 Feasibility and safety of the alloxan‐induced DRN rabbit model (80 mg/kg) over 9 weeks post‐induction

#### 3.2.1 Effect on diabetic markers and lipid profile in the alloxan‐induced model (80 mg/kg)

**FIGURE 4:**
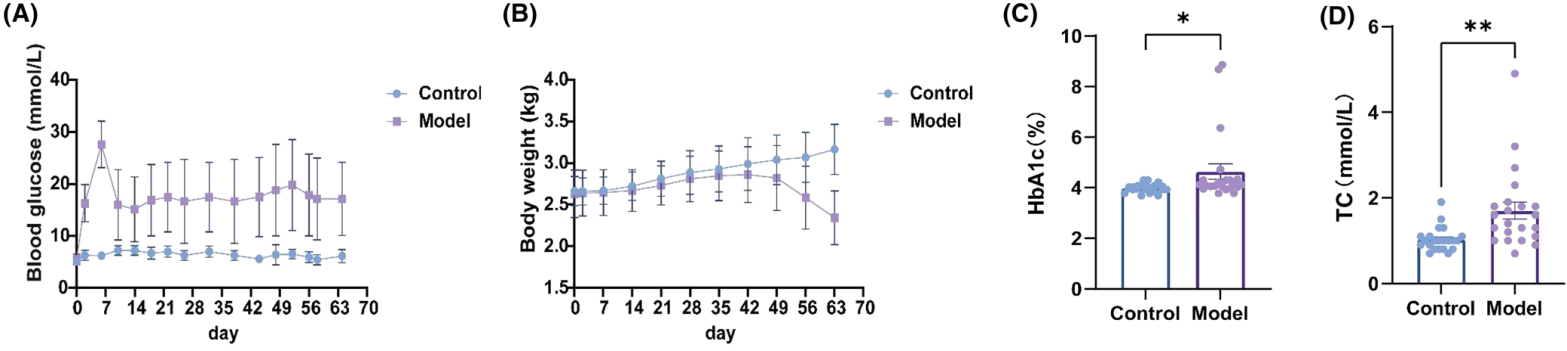
General observation of the alloxan‐induced diabetic rabbit model over the 9‐week post‐induction period. (A) Quantitative analysis demonstrated that BGLs in alloxan‐treated rabbits were higher than those in the control group. (B) Quantitative analysis revealed that blood weight was lower in alloxan‐treated rabbits compared to the control group. (C) Quantitative analysis indicated that HbA1c levels were elevated in alloxan‐induced rabbits. (D) Quantitative analysis demonstrated that TC levels were increased in alloxan‐induced rabbits. Data are expressed as the mean ± SD (*n* = 22 rabbits per group). *p < 0.05, **p < 0.01.

Each rabbit in the model group received a single alloxan injection at a dose of 80 mg/kg (*n* = 22). Changes in final body mass, blood glucose concentrations, HbA1c levels, and serum fasting TC were evaluated in model and control groups, with outcomes illustrated in Figure 4. Statistical analysis indicated that the model group (80 mg/kg) exhibited significantly elevated BGLs compared to the control group (Figure 4A). During 9 weeks following alloxan administration, the mean body weights of the control group remained statistically unchanged, whereas the model group (80 mg/kg) exhibited a significant reduction (*p* < 0.05) (Figure 4B). HbA1c levels in the model group increased to 4.994% ± 0.5479%, notably higher than the 4.039% ± 0.039% recorded in the control group (Figure 4C). Additionally, TC levels in the model (80 mg/kg) group were significantly elevated relative to the control group (*p* < 0.001) (Figure 4D). Elevated BGLs and disrupted lipid metabolism were key factors in the development of DRN.^45^ This study established a diabetic rabbit model to closely replicate disease characteristics observed in patients.

#### 3.2.2 Functional impairments in the DRN model (80 mg/kg) detected by ERG and VEP

**FIGURE 5:**
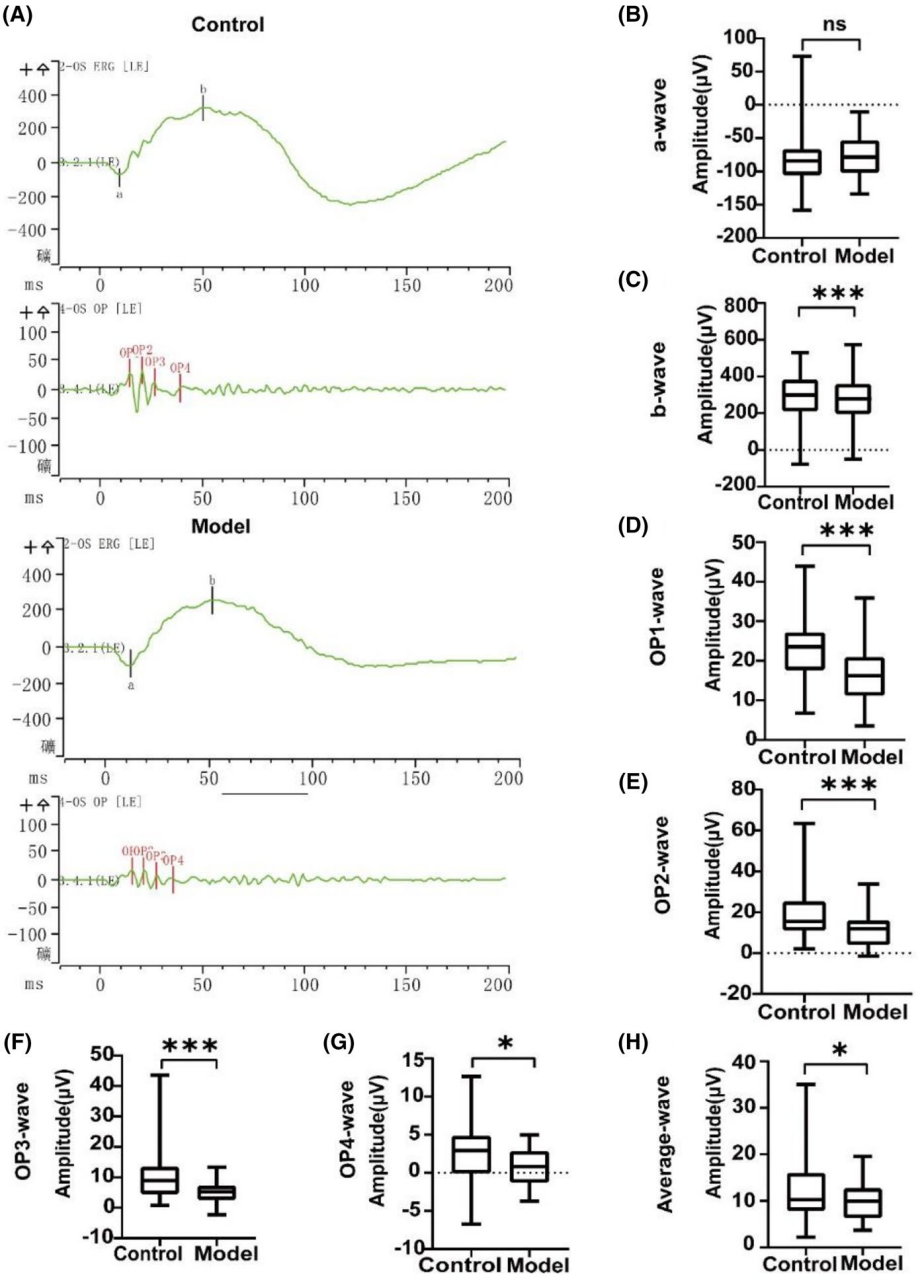
Graphical representation of dark‐adapted 3.0 ERG amplitudes in the model group compared to the control group during 9 weeks post‐induction. (A) Representative ERG waveform of control and model groups. (B) Comparison of the mean amplitude of a‐wave. (C) Comparison of the mean amplitude of b‐wave. (D) Comparison of the mean amplitude of OP1 wave. (E) Comparison of the mean amplitude of OP2 wave. (F) Comparison of the mean amplitude of OP3 wave. (G) Comparison of the mean amplitude of OP4 wave. (H) Comparison of the mean amplitude of the average of all waves. Significant lower amplitudes were observed in the model group compared to the control group. OP1: Oscillatory potential wave 1; OP2: Oscillatory potential wave 2; OP3: Oscillatory potential wave 3; OP4: Oscillatory potential wave 4. Average represents the mean amplitudes of all OP waves measured. Data are expressed as the mean ± SD (*n* = 22 rabbits per group). *p < 0.05, **p < 0.001,***p < 0.0001.

**FIGURE 6:**
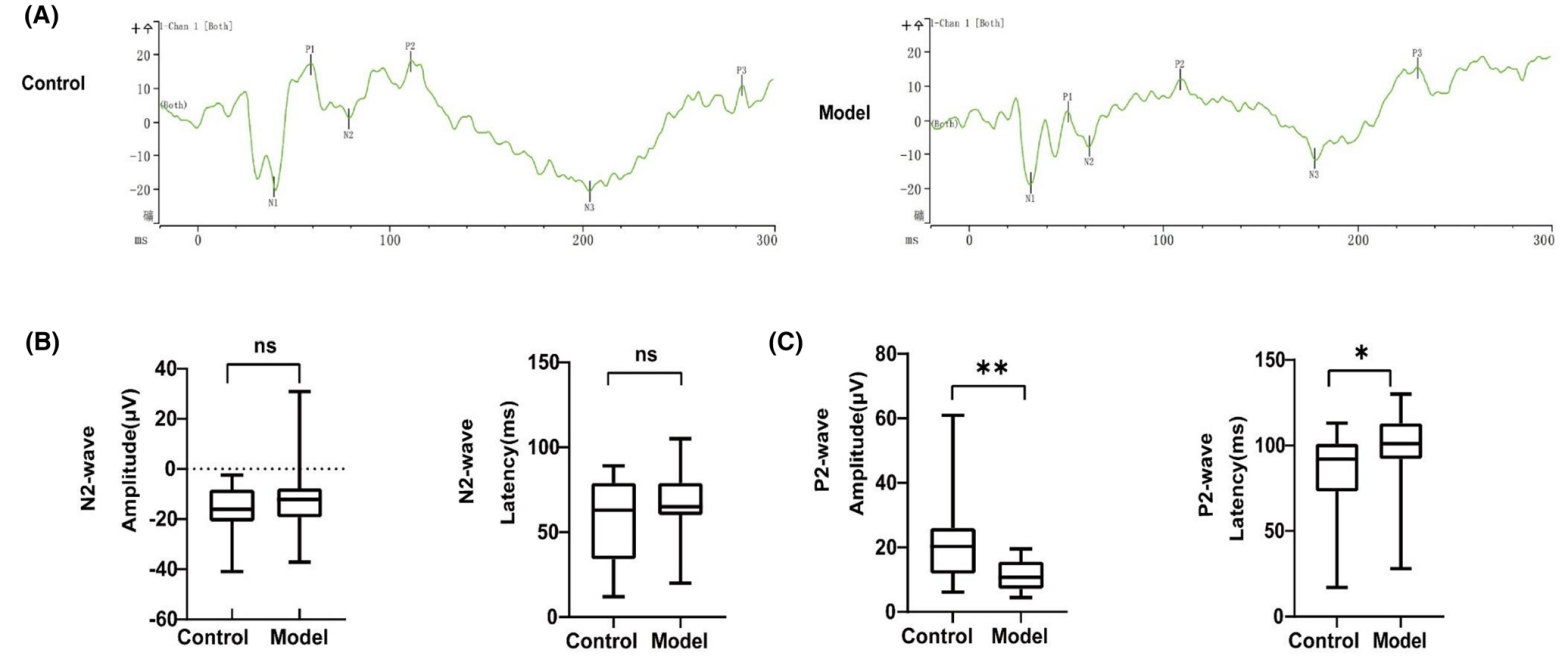
VEPs of the control and model groups were compared during the 9‐week post‐induction period. (A) Representative waveform graphs of control and model groups. (B) Comparison of the mean amplitude and latency of N2 wave. (C) Comparison of the mean amplitude and latency of P2 wave. Significant reductions in the mean amplitude were observed in the model group compared to the control group. Peak refers to the light peak. Data are presented as the mean ± SD (*n* = 22 rabbits per group).*p < 0.05, **p < 0.01.

ERG and VEP provide detailed insights into the visual function and the potential effects of diabetes on its integrity.^46^ VEPs reflect changes in the bioelectric potentials of the occipital cortex elicited by visual stimuli.^47^ Following anesthesia, rabbits underwent dark‐adapted and light‐adapted ERG recordings, including OPs. ERG and OP patterns remained consistent across three consecutive experiments. Representative ERG waveforms obtained from control and model (80 mg/kg) rabbit eyes at 9 weeks post‐alloxan injection are depicted in Figure 5A and Figures S1A and S2A. Reduced a‐wave amplitudes indicated photoreceptor damage and impairments of retinal physiological function.^48^ The light‐adapted 3.0 ERG a‐wave demonstrated a significant increase in the 80 mg/kg model group relative to controls (*p* < 0.05; Figure S1B), whereas no intergroup differences were observed in the dark‐adapted 3.0 ERG (Figure 5B) or dark‐adapted 10.0 ERG a‐wave amplitudes (Figure S2B). The b‐wave reflects the electrical activity of the inner nuclear layer (INL).^49^ In the model (80 mg/kg) group, the b‐wave amplitudes of the dark‐adapted 3.0 ERG (*p* < 0.001), light‐adapted 3.0 ERG (*p* < 0.001), and dark‐adapted 10.0 ERG (*p* = 0.027) were significantly reduced (Figure 5C, Figures S1C and S2C), while the peak‐wave amplitudes of the light‐adapted 3.0 ERG did not change (Figure S1D). Altered OP wave patterns were observed during early investigations of DR, including studies involving human and animal subjects.^50^, ^51^, ^52^ In the model group (80 mg/kg), the dark‐adapted 3.0 ERG revealed a significant reduction in OP wave amplitudes (*p* < 0.05) (Figure 5D–H). Additionally, the OP analysis identified a significant decrease in the amplitude of the OP4 wave under dark‐adapted 10.0 ERG conditions (Figure S2G), the other OP waves showed no change (Figure S2D–F,H). At 9 weeks post‐alloxan injection, increased VEP responses were observed in the model groups (Figure 6A). Previous studies suggest that a transient luminance F‐VEP waveform primarily originates from activity within the striate and extrastriate cortex.^53^, ^54^, ^55^ Significant differences in P2 latency (*p* = 0.046) and P2 amplitude (*p* < 0.001) were observed between the control and model (80 mg/kg) groups (Figure 6B,C).

#### 3.2.3 Minimal retinal alterations were observed via structural imaging in the DRN model (80 mg/kg alloxan)

##### Optos UWF pseudocolor imaging and retinal vascular abnormalities assessed by WF‐FFA

**FIGURE 7:**
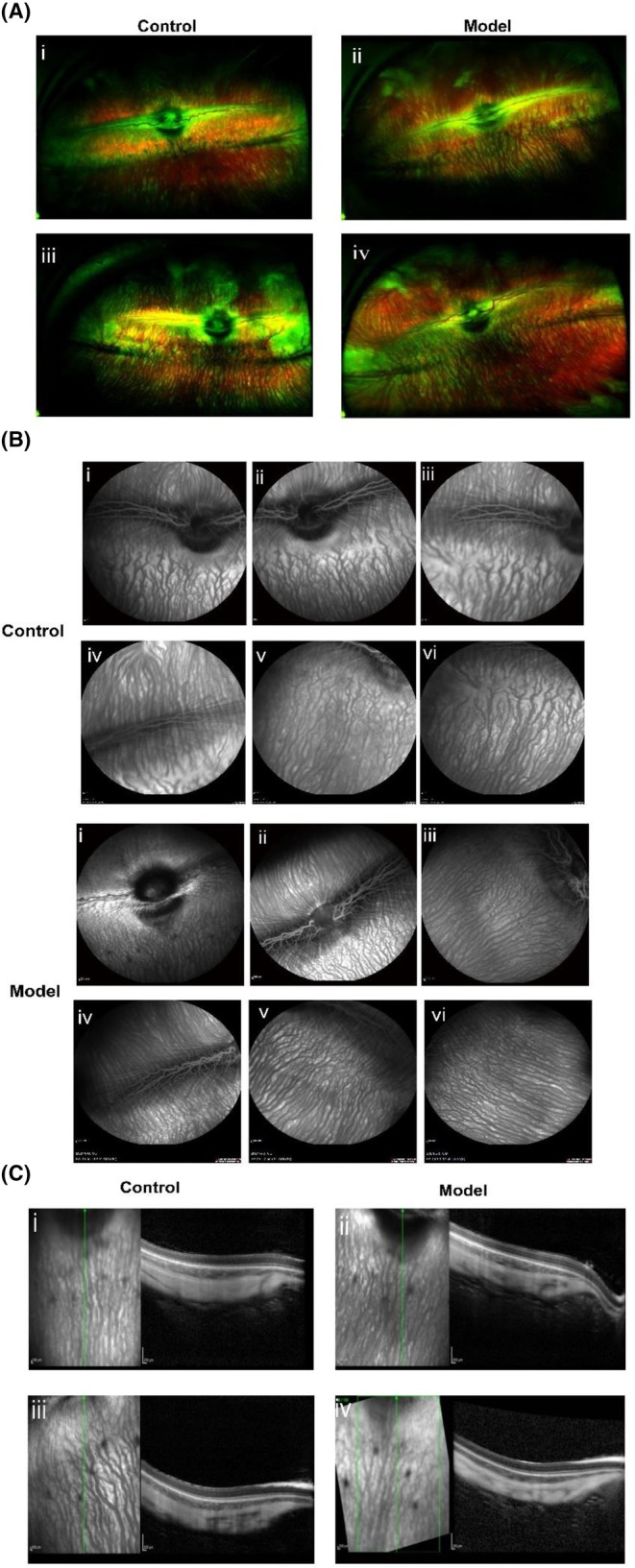
Optos UWF, SD‐OCT, and WF‐FFA results in control and model groups during the 9‐week post‐induction period. (A) Optos UWF images: (i) and (ii) represent the left and right eyes of normal rabbits, and (iii) and (iv) indicate the left and right eyes of 8‐week adult diabetic model rabbits. (B) FFA images: Control group represents normal rabbits, and DRN group indicates 8‐week adult diabetic model rabbits. (C) SD‐OCT images: (i) and (ii) represent control rabbits, and (iii) and (iv) represent 8‐week diabetic model rabbits.

The optic disc of the fundus appeared oval, larger than the human optic disc, and measured approximately 3–4 mm in width. Two broad, white, raised, and opaque nerve fiber bundles were evident within the medulla. The nasal and temporal branches of the central retinal artery aligned with these medullary nerve fiber bundles, while no additional vascular branches were observed elsewhere in the retina. Prominent choroidal macrovessels were observed, accompanied by a denser underlying vascular network, indicating a significant difference in retinal blood flow between rabbit and human eyes. A slightly darker band, approximately 1–3 mm wide, was observed beneath the medullary nerve fibers, delineating the optic zone region in rabbits (Figure 7A). Quantitative analysis revealed no significant difference between DRN and Control groups (*p* > 0.5). No signs of DRN‐related alterations, including retinal hemorrhages, exudates, and microangiomas, were observed in the model rabbits. WF‐FFA provides a more comprehensive assessment of microvascular abnormalities, retinal non‐perfusion zones, neovascularization, vascular fluorescein leakage, and perfusion dynamics than fundoscopy or color imaging alone, thereby enhancing the understanding and evaluation of retinal vascular disorders by ophthalmologists.^56^ WF‐FFA images from Control and DNR groups revealed no evidence of microvascular abnormalities, fluorescein leakage, perfusion defects, or scleral buckle leakage. The images demonstrated normal microvascular architecture without dilation or distortion, accompanied by uniform blood flow and an intact blood‐retinal barrier. The absence of fluorescein leakage further confirms the preservation of vascular wall integrity and adequate retinal and choroidal perfusion (Figure 7B).

##### Visualization of retinal structures

SD‐OCT enabled the visualization of distinct retinal layers, including the ganglion cells layer (GCL), inner plexiform layer (IPL), INL, outer plexiform layer (OPL), outer nuclear layer (ONL), photoreceptor layer (PRL), and choroid (Figure 7C). Retinal OCT images from model rabbit eyes were structurally normal compared to those of control eyes.

**FIGURE 8:**
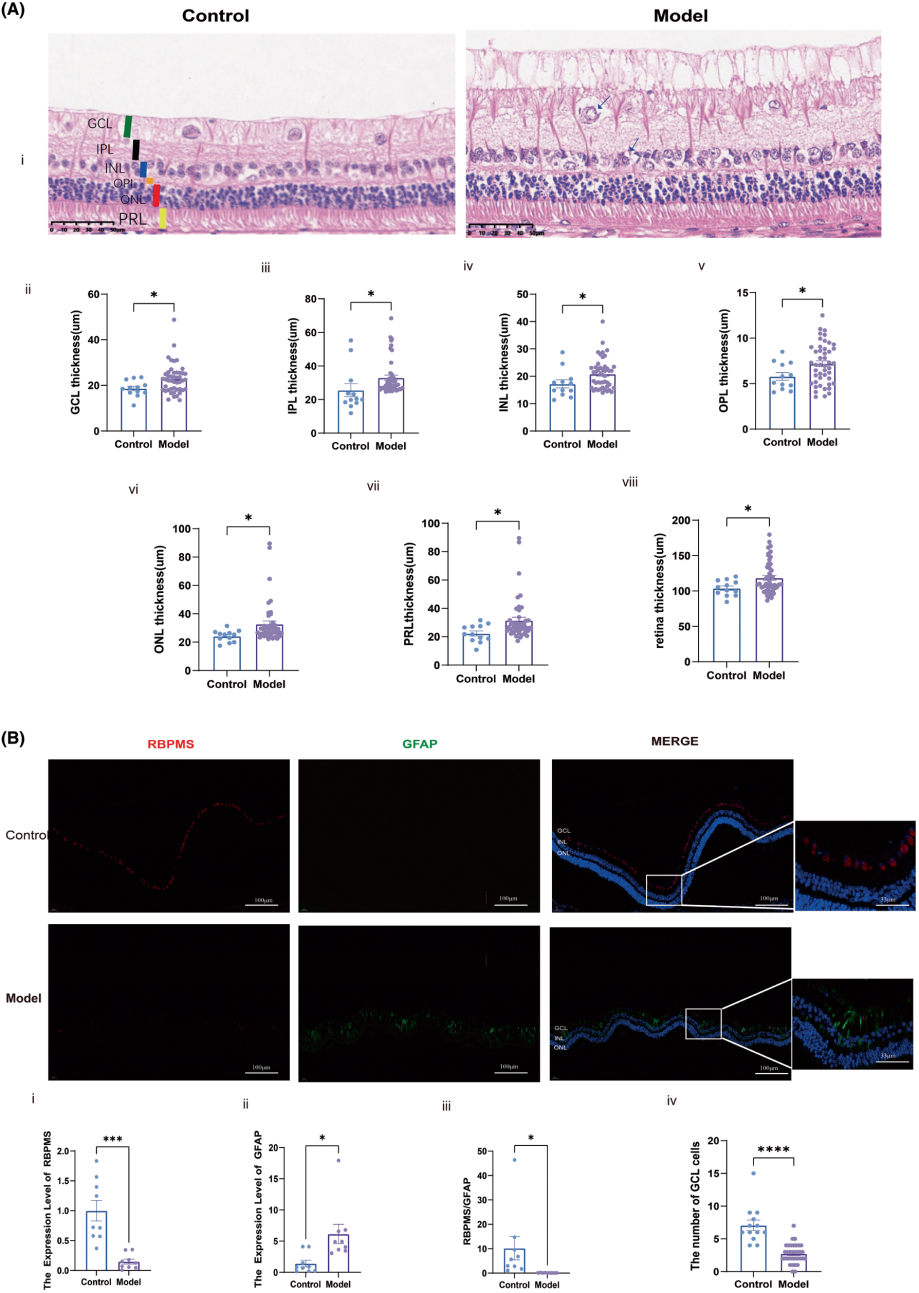
Representative retinal tissue sections stained with H&E from normal and DR rabbits during the 9‐week post‐induction period. (A) H&E‐stained retina tissue sections from control and model rabbits exhibiting retina thickness under a light microscope at 400× magnification. (B) Expression of RBPMS and GFAP in the retina was assessed using IF staining. GCL, Ganglion cell layer; INL, Inner nuclear layer; IPL, Inner plexiform layer; ONL, Outer nuclear layer; OPL, Outer plexiform layer; PRL, Photoreceptor cell layer. *p < 0.05, ***p < 0.0001, ****p < 0.0001.

Although the model group demonstrated reduced retinal thickness in superior and inferior regions and a slight increase in inferior choroidal thickness relative to controls, these variations were statistically non‐significant (all *p* > 0.05). These findings indicate that 9 weeks of full oxygen acid treatment did not induce notable pathological alterations in ocular structure, providing a stable baseline for subsequent investigations (Table S1). The rabbit retina did not display a concave macular center. Retinal thickness varied across regions, with thinner measurements observed superiorly compared to inferiorly and the greatest thickness beneath the optic disc. Choroidal thickness also varied across different regions, with a notably thinner layer surrounding the optic disc that gradually increased in thickness with distance from the optic disc, reaching greater values superiorly and inferiorly. Extremely thin retinal and choroidal tissue layers were observed in regions corresponding to myelinated nerve fiber reflex areas (Figure S3A–F). A histomorphological analysis of retinal tissue was conducted using H&E staining to fully assess the structural alterations following retinal injury. Representative retinal sections along the vertical meridian through the optic nerve head from the control and model (80 mg/kg) groups at 9 weeks post‐alloxan injection are presented in Figure 8A. As illustrated in Figure 8A, the inner retinal thickness in the model group (80 mg/kg), including the GCL, IPL, INL, OPL, ONL, and PRL, was significantly increased compared to the control group (*p* < 0.05). Enlarged cellular tissue gaps were observed across all retinal layers, with pronounced interlayer edema extending from IPL to GCL in the model group (80 mg/kg). The cellular gaps between IPL and GCL appeared edematous, loosely arranged, and disorganized, while some cells within the INL demonstrated vacuolated degeneration.

#### 3.2.4 Glial activation and neuronal loss in diabetic retinas

Aberrant activation of glial cells, including microglia and astrocytes, plays a central role in neuroinflammation associated with DNR. A significant 50% increase in GFAP‐positive glial cells was observed in the model group (80 mg/kg) compared to controls (*p* < 0.05) (Figure 8B), with GFAP‐positive fibers extending across all retinal layers, consistent with the activation of Muller glia. Furthermore, alterations in ganglion cell signaling in DR appear to correlate with ganglion cell loss. IF staining identified distinct labeling of ganglion cells using RBPMS and glial cells with GFAP within the GCL without overlap between the two markers. The analysis revealed a marked reduction in RBPMS‐labeled ganglion cell density, displaced GABAergic amacrine cell density, and total GCL cell density in the model group compared to the control group. A smaller population of unlabeled presumed ganglion cells was also detected (*p* < 0.001).

## 4 DISCUSSION

DRN, the primary manifestation of ocular diabetic damage, is characterized by dysfunction of retinal neurons and glial cells.^16^, ^17^ Hyperglycemia initiates a cascade of biochemical events that lead to early neuronal apoptosis and glial activation.^34^ These neuroretinal changes emerge before the onset of clinical DR in individuals with diabetes and precede DR microvascular pathology in diabetic mouse models.^57^ However, a significant limitation of diabetic rabbit models remains the high mortality rate among experimental animals.^16^, ^17^

This study identified that a single 80 mg/kg injection of alloxan resulted in a low mortality rate and a high modeling success rate, indicating its suitability as an optimal dose for establishing diabetic rabbit models. Currently, no reports provide a standardized optimal dosage of alloxan for diabetes induction in rabbits. A notable increase in HbA1c levels was observed in diabetic rabbits following a single intravenous alloxan injection (80 mg/kg). Insulin deficiency caused by pancreatic beta‐cell destruction remains a defining feature of diabetes. HbA1c serves as a critical diagnostic indicator for diabetes and is regarded as more reliable than glucose levels, as it remains elevated despite fluctuations in BGLs in patients with diabetes. Consistent with these findings, Bem et al.^58^ reported a similar increase in HbA1c levels in alloxan‐induced diabetic rabbits over 10 weeks. The developed model exhibited sustained hyperglycemia accompanied by a modest elevation in triglyceride levels. Dyslipidemia appeared at an early stage and was aligned with the pattern of diabetic dyslipidemia observed at week 6. Helfenstein et al.^16^ demonstrated that rabbits with diet‐induced impaired glucose tolerance developed hypercholesterolemia by week 24. In contrast, the diabetic rabbit model was developed through a high‐sugar, high‐fat diet, resulting in a distinct pathophysiological profile. Hyperglycemia, lipid metabolism disorders, and abnormalities in the insulin signaling pathway are widely recognized as the initiating factors underlying various pathophysiological changes in DRN (Figure 3A–D). Notably, progressive retinal changes in DRN are closely associated with elevated levels of HbA1C and TC.^58^, ^59^ These findings suggest the successful establishment of the rabbit model replicating key features of DRN.

DRN leads to retinal abnormalities that impair function before the onset of clinical DR, in some cases, even before diabetes is diagnosed.^17^, ^60^ In this study, a significant reduction in mean b‐wave amplitude was identified in the model rabbits (*p* < 0.01), accompanied by a decrease in the amplitude of the OP wave (Figure 5). This reduction in amplitude strongly correlates with the extent of retinal damage,^5^ suggesting delayed signal transmission in bipolar cells and Muller cell dysfunction.^61^ These findings are consistent with previous studies involving streptozotocin‐induced diabetic rats and further support electrophysiological impairments as an early indicator of retinal neuropathy.^62^ VEP provides an objective evaluation of visual system function.^53^ In model rabbits, a significant reduction in mean P2 wave amplitudes (*p* < 0.05) and a prolongation in P2 wave latency were observed (Figure 6), indicating optic nerve injury and disruption of the visual pathway. A decrease in amplitude combined with increased latency in VEP readings strongly suggests damage to the optic nerve. Miura^54^ demonstrated that patients with diabetes mellitus may exhibit antecedent neuropathy despite the absence of clinically detectable ophthalmoscopic or morphological abnormalities. Moreover, VEP can detect such functional impairments before the appearance of visible changes during fundus examination. These findings are consistent with the results of this study. The present study represents the first assessment of visual impairment in DRN model rabbits using full‐field ERG and VEP recordings under scotopic and photopic conditions. The results suggest that early retinal dysfunction and retinopathy in DRN may initially manifest in the optic retina and central optic nerve.

Structural analyses through SD‐OCT and H&E staining provided complementary perspectives on retinal alterations in early diabetic rabbits. SD‐OCT was performed on conscious animals to assess retinal and choroidal thickness in superior and inferior regions (Figure 7B). At 9 weeks, diabetic rabbits exhibited no significant retinal thinning compared to controls (Table S1). These findings align with the observations in a diabetic mouse model, which demonstrate impairments in a‐wave, b‐wave, and OPs despite preserved inner retinal thickness.^63^ This evidence underscores the suitability of the DRN rabbit model for studying early neural retinal pathology while highlighting the advantage of OCT in awake animals to reduce use and support animal welfare.

In contrast, H&E staining revealed marked retinal edema in diabetic animals, characterized by disorganized cellular distribution from the retinal nuclear layer to the ganglion cell layer, reduced intercellular spacing, and increased vacuolization (Figure 8).^64^, ^65^ Comparable disruptions have been observed in STZ‐induced diabetic rats, including alterations in GCL, INL, and ONL at 8 weeks^66^ and vacuolar degeneration of ganglion cells at 4 weeks.^67^ Notably, histologically measured retinal thickness was significantly greater than that obtained via OCT, likely due to inherent differences between in vivo imaging and ex vivo histological processing. In vivo OCT, despite its high‐resolution capabilities, may underestimate diffuse edema because its resolution and segmentation algorithms—primarily optimized for human retinal architecture—are not fully attuned to detect subtle, uniformly distributed changes, particularly in rabbit models with distinct retinal layer definitions. Conversely, H&E staining of ex vivo sections offers exquisite cellular and subcellular detail but is susceptible to processing artifacts, such as those arising from fixation, dehydration, and embedding, which can exaggerate tissue swelling. Consequently, the full‐thickness edema observed histologically likely reflects both genuine pathological alterations and artifacts introduced during tissue processing, whereas OCT yields a more conservative estimate of retinal thickness. Future research should aim to refine OCT segmentation protocols and develop species‐specific criteria to enhance the correlation between these imaging modalities.

Furthermore, WF‐FFA and Optos UWF imaging revealed no vascular abnormalities or basement membrane thickening in the DRN model, with similar results between diabetic and control groups (Figure 7). Specifically, FFA is highly sensitive for detecting microvascular leakage, subtle perfusion defects, and early vascular abnormalities. By dynamically imaging the distribution of fluorescein dye in the retinal vasculature, FFA provides detailed visualization of vascular integrity, enabling the identification of even minor instances of leakage that might be missed by other modalities.^68^ Although Ljubimov et al.^69^ reported basement membrane thickening in diabetic retinopathy, such alterations were absent in our model. Notably, this study is the first to assess early DRN severity in rabbits using Optos UWF imaging, which, along with SD‐OCT, confirmed the presence of a well‐defined visual streak and clear optic bands in awake diabetic rabbits, supporting findings by Paulus et al.^70^

This study identified pronounced glial activation and substantial neuronal loss in DRN retinas. This activation may intensify retinal damage by amplifying inflammatory signaling pathways, aligning with prior findings that associate glial activation with DRN.^71^, ^72^ Concurrently, the results revealed a marked reduction in ganglion and displaced amacrine cells within the GCL. Decreased RBPMS‐labeled ganglion cell density further supports the hypothesis that diabetic retinas undergo progressive neuronal loss, ultimately disrupting visual signaling.

This model effectively isolates early neurodegenerative events from subsequent microvascular pathology, establishing it as a valuable preclinical tool for investigating the mechanisms underlying DRN. Full‐field ERG and VEP emerged as highly sensitive screening tools, capable of detecting retinal nerve dysfunction before the onset of irreversible damage occurs. While early neuronal and glial alterations are evident in preclinical diabetes models, conventional diagnostic approaches typically identify neuropathy only during more advanced stages. Preclinical evidence indicates that retinal neuropathy, partially characterized by photoreceptor dysfunction, precedes overt vascular alterations, reinforcing the rationale for neuroprotective strategies to prevent or delay apoptotic neuronal cell death. These findings highlight the critical need for early detection and intervention targeting neurodegenerative processes in DR. In summary, this model elucidates the temporal progression of diabetic retinal alterations while emphasizing the therapeutic potential of early neuroprotective approaches to enhance clinical outcomes in individuals with diabetes.

This study has some limitations. First, using a rabbit model introduces physiological and anatomical disparities compared to humans, potentially limiting direct clinical applicability. Although the DRN model was successfully replicated, the short observation period restricted the comprehensive assessment of disease progression. Second, suboptimal housing conditions, including limited control over movement and dietary intake following induction, may have introduced confounding factors. Alloxan injection (80 mg/kg) yielded promising outcomes; however, the small sample size constrains statistical robustness and reproducibility. Future studies should employ larger cohorts and adopt a multidisciplinary strategy integrating in vitro, ex vivo, and clinical research to more precisely define the biological and pathophysiological mechanisms underlying DRN.

## 5 CONCLUSION

An 80 mg/kg dose of alloxan was identified as optimal and safe for establishing a DRN model in rabbits. Multimodal evidence from ERG, VEP, H&E, and IF, combined with the absence of microvascular alterations at 9 weeks, validates the model and accurately replicates the pathophysiological features observed in clinical DRN. Notably, this integrated analysis represents a novel approach, as ERG with OPs and VEP responses demonstrated greater sensitivity to early retinal alterations than OCT and UWF imaging. These findings highlight the significant impact of early hyperglycemia on the retinal neural network and establish a strong foundation for advancing DRN research and developing targeted therapeutic interventions.

## AUTHOR CONTRIBUTIONS

**Xinlu Li:** Conceptualization; data curation; writing – original draft; writing – review and editing. **Xiaojing Dong:** Methodology; resources; writing – original draft; writing – review and editing. **Defei Feng:** Conceptualization; writing – original draft; writing – review and editing. **Bai Li:** Resources; writing – original draft; writing – review and editing. **Han Hu:** Conceptualization; data curation; software; writing – review and editing. **Wei He:** Conceptualization; funding acquisition; writing – review and editing. **Zhongjian Liu:** Data curation; formal analysis; writing – original draft. **Chenchen Huang:** Conceptualization; project administration; writing – review and editing. **Zhizhou Shi:** Supervision; validation; writing – original draft. **Yan Mei:** Funding acquisition; resources; writing – original draft; writing – review and editing.

## FUNDING INFORMATION

This study was funded by the Key Project of Joint Special Funds for Applied Basic Research of Yunnan Provincial Department of Science and Technology Kunming Medical University (Grant No. 2018FE001(‐180)), the Clinical Research Center of the First People's Hospital of Yunnan Province (Grant No. 2023YJZX‐LN01), “the Kunming University of Science and Technology School of Medicine Postgraduate Innovation Fund,” the Research Plan of the National Natural Science Foundation of China (82460210), and the Provincial Key Clinical Specialty Platform of the First People's Hospital of Yunnan Province (Grant No. 2024EKKFKT‐04).

## CONFLICT OF INTEREST STATEMENT

The authors declare that the research was conducted without any commercial or financial relationships that could be interpreted as a potential conflict of interest.

## ETHICS STATEMENT

Ethics approval was obtained from the Committee of Ethics in Animal Experimentation of Kunming Medical University, Yunnan Province, China, consistent with the Use of Animals in Ophthalmic and Vision Research (Ethical Review No.: kmmu20221504).

## Supporting information


Appendix S1.
