# Supplementary material for: Establishment of a novel alloxan‐induced rabbit model exhibiting unique diabetic retinal neuropathy features assessed via ERG + VEP
Source: Animal Model Exp Med. 2025 Jun 19;8(9):1552–66. doi: 10.1002/ame2.70032 (PMC12710098; doi:10.1002/ame2.70032)
Supplement: Supplementary file 1 — Appendix S1. [file AME2-8-1552-s001.docx]

Supplementary Material

Establishment of a New Alloxan-Induced Rabbit Model with Unique Diabetic Retinal Neuropathy Features Assessed by ERG+VEP

Xinlu Li*

*** Correspondence:** Yan Mei: meikm@163.com

# Supplementary Figures and Tables

## Supplementary Figures


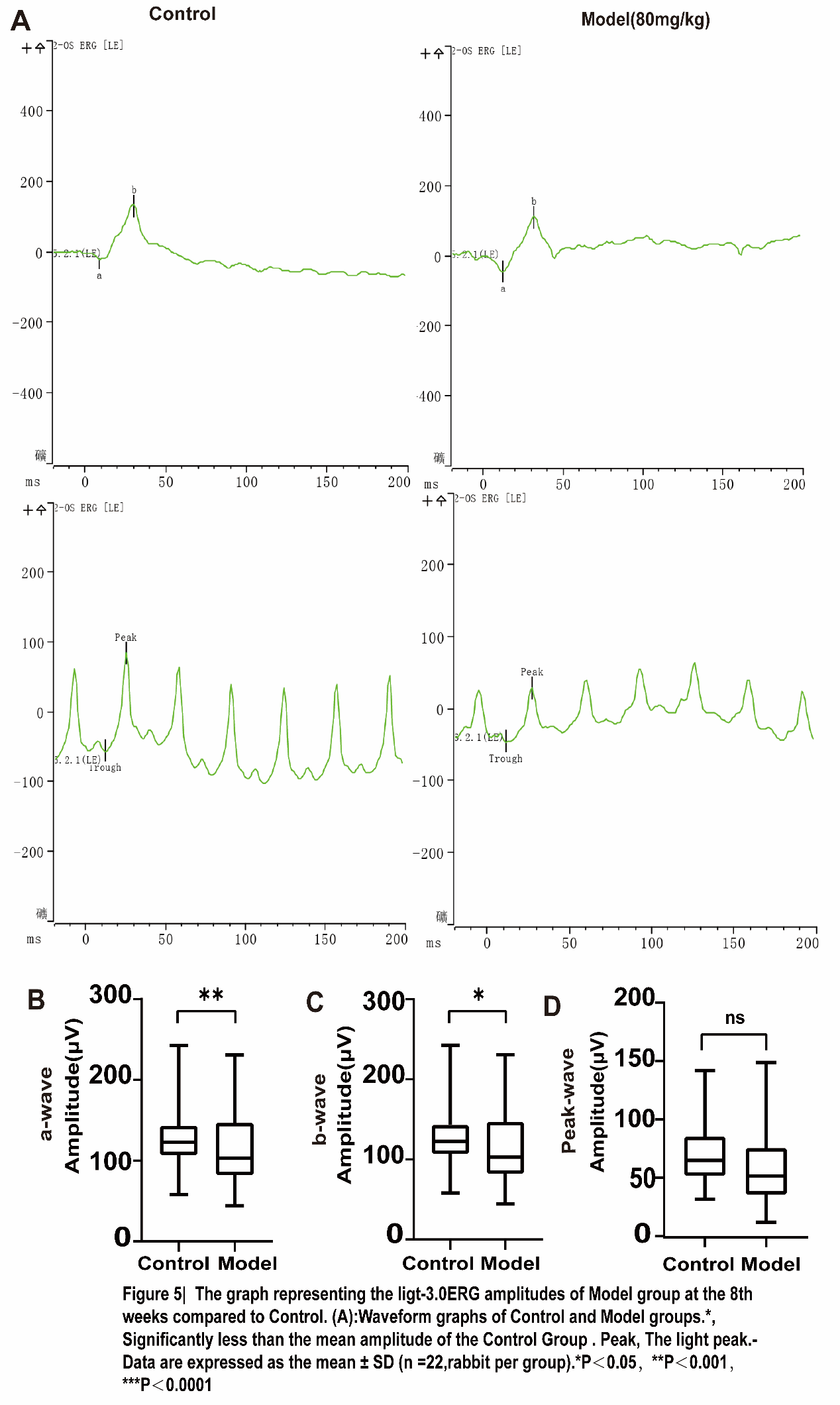


**Supplementary Figure 1 The graph representing the ligt-3.0ERG amplitudes of Model group after 9 weeks of alloxan injection compared to Control.** (A): Waveform graphs of Control and Model groups. (B)Comparison of the mean amplitude of "a" wave. (C)Comparison of the mean amplitude of "b" wave. (D)Comparison of the mean amplitude of "Peak" wave.*, Significantly less than the mean amplitude of the Control Group . Peak, The light peak. Data are expressed as the mean ± SD (n =22,rabbit per group).*P＜0.05，**P＜0.001


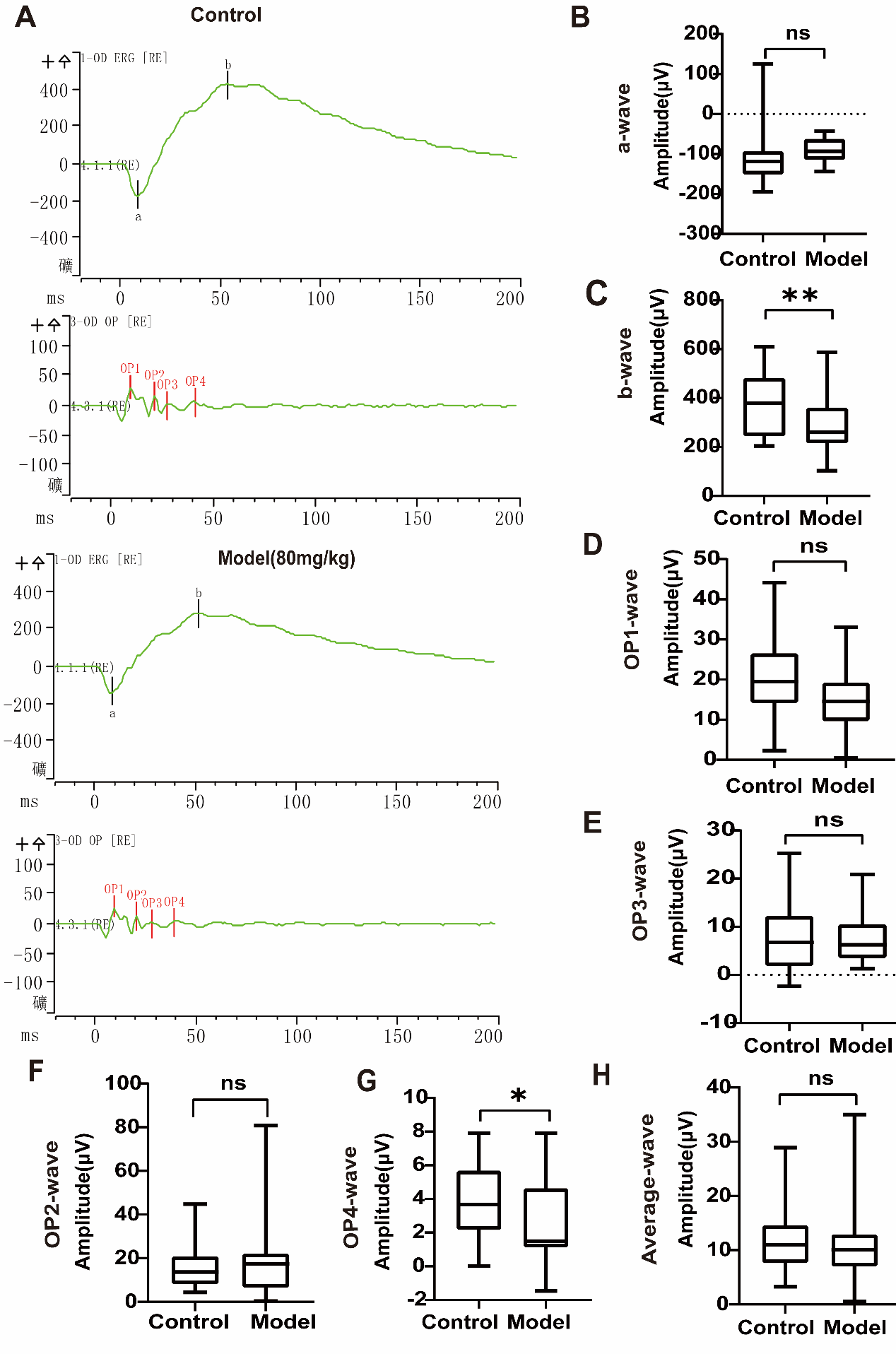


**Supplementary Figure 2 The graph representing the dark-10.0ERG amplitudes of Model group after 9 weeks of alloxan injection compared to Control.** (A)Waveform graphs of Control and Model groups .(B)Comparison of the mean amplitude of "a"wave.(C)Comparison of the mean amplitude of "b"wave.(D)Comparison of the mean amplitude of "OP1"wave.(E)Comparison of the mean amplitude of "OP2"wave.(F)Comparison of the mean amplitude of "OP3"wave.(G)Comparison of the mean amplitude of "OP4"wave.(H)Comparison of the mean amplitude of "Average"wave.*, Significantly less than the mean amplitude of the Control Group OP1, Oscillatory Potential Wave 1; OP2, Oscillatory Potential Wave 2; OP3, Oscillatory Potential Wave 3; OP4, Oscillatory Potential Wave 4. Average, The average of all OP waves at measurement. Data are expressed as the mean ± SD (n =22, rabbit per group). *P＜0.05，**P＜0.001


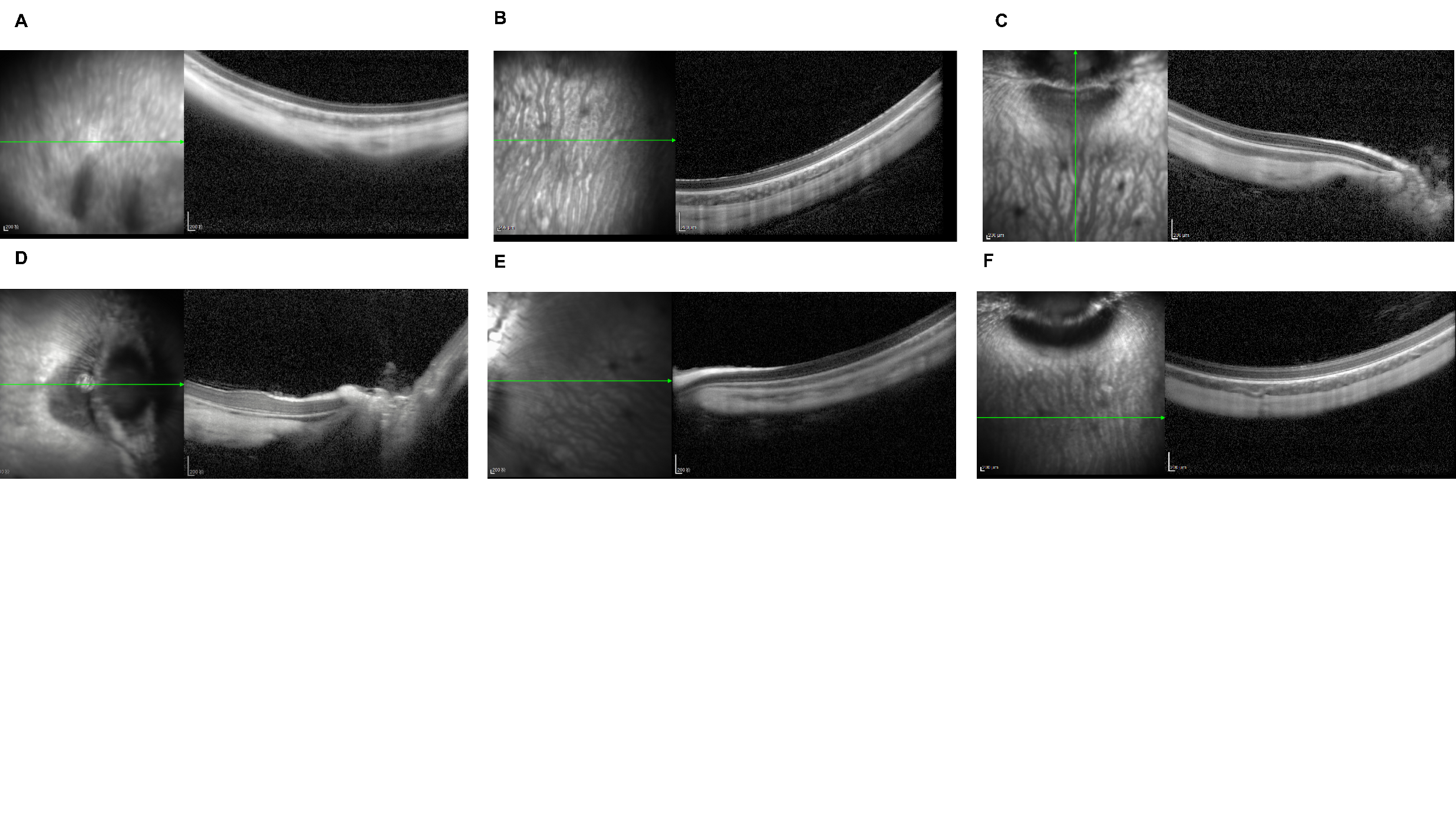


**Supplementary Figure** **3 The retina of a normal rabbit.** (A) superior retina. (B) inferior retina. (C) retinal and choroidal layers are present at the location of the myelinated nerve fiber reflex. area. (D) Peripapillary peripheral choroid. (E) Gradual thickening of the choroid. (F) The thicker choroid underneath.nerve fiber reflex areas.

## Supplementary Tables

**Supplementary Table.1 SD-OCT results were obtained at Control and Model group after 9 weeks of alloxan injection**

| **Project**  **parameters** | **Upper Retina Thickness(μm)** | **Lower Retina**  **Thickness(μm)** | | **Lower Choroid Thickness(μm)** | **Optic papilla upper and lower diameters(mm)** | **anteroposterior diameter of the optic papilla**  **(mm)** | **Optic papilla**  **area(mm^2^)** |
| --- | --- | --- | --- | --- | --- | --- | --- |
| Control | 156.75±8.08 | | 195.62±7.45 | 95.57±14.03 | 4.31±0.26 | 4.60±0.15 | 15.38±3.16 |
| Model | 146.38±2.83 | | 181.62±2.60 | 104.14±12.27 | 3.85±0.52 | 4.42±0.77 | 14.41±2.67 |
| t | 1.21 | | 1.77 | -2.18 | 0.784 | 0.59 | 0.33 |
| *P* | 0.246 | | 0.111 | 0.072 | 0.450 | 0.571 | 0.745 |
